# Survey data reflecting popular opinions of the causes and mitigation of climate change

**DOI:** 10.1016/j.dib.2017.07.060

**Published:** 2017-07-27

**Authors:** Jonathan E. Thompson

**Affiliations:** Department of Chemistry, Texas Tech University, Lubbock, TX 79409-1061, United States

**Keywords:** Climate change, Public opinions, Geo-engineering, Greenhouse gases, Aerosols

## Abstract

The data presented within this manuscript reports the results of a 20-question opinion survey concerning popular beliefs regarding the causes of and possible mitigation of climate change. The results and opinions from 746 survey respondents are presented. The data reflects certain misconceptions of climate change, and is useful for investigators to begin forming opinions of the public's knowledge regarding the potentially inflammatory topics of climate change, greenhouse gases, and geo-engineering.

**Specifications Table**

**Table:** 

Subject area	*Environmental Science, Public Policy, Public Opinion*
More specific subject area	*Climate Change*
Type of data	*Opinion Survey Results in Tables & Lists*
How data was acquired	*Online Survey*
Data format	*Summarized Responses for Each Survey Question is Reported*
Experimental factors	*Various*
Experimental features	*An online survey was administered through Google forms to sample opinions related to climate change. Participants were recruited through social media, University email lists, and Amazon Mechanical Turk.*
Data source location	*Online Survey – various locations*
Data accessibility	*Data is provided within this article*

**Value of the data**

• The data allows investigators to have a base understanding of current public opinion and knowledge regarding the topic of climate change.
• As the data reports some scientific misconceptions it will allow targeted educational campaigns to be established to contribute to the public good.
• The data establishes current public beliefs and concerns that serve as a baseline for future study and establishment of public policy.

## 1 Data

Below I report the quantitative survey responses that describe public knowledge / opinion regarding climate change. It should be noted that the responses should not be considered scientifically accurate. Rather, the data reported here reflects only public opinion, not scientific facts.

*Response Data for Question 1. Self reported age range.*

**Table:** 

***Age range***	***% of Total respondents (N = 746)***
*18–30*	*43.6*
*31–40*	*30*
*41–50*	*12.7*
*51–60*	*7.2*
*61–70*	*5.3*
*71–80*	*0.8*
*Over 80*	*0.4*

*Response Data for Question 2. Self Reported Nationality.*

**Table:** 

***Self-Reported Nationality***	***# of Respondents***	***% of Total Respondents (N = 746)***
*Austrian, Bahreini, Bangladeshi, Chilean, Ecuadorian, Finnish, Greek, Guatemalan, Honhuran, Luxenbourgish, Macedonian, Nepali, Netherlands, Romanian, Serbian, Singaporean, Slovene, South African, Spanish, Swedish, Swiss, Turkish, Vietnamese, Yemeni, Zambian*	*1 each*	*0.13 each*
*Brazillian, Canadian, Chinese, French, Jamaican, Korean, Malaysian, Nigerian, Thai,*	*2 each*	*0.27 each*
*Irish, Mexican, Native American*	*3 each*	*0.4 each*
*British, Colombian, Filipino, German, Italian,*	*4 each*	*0.54 each*
*Asian*	*5 each*	*0.67*
*Venezuelan*	*8*	*1.07*
*Hispanic*	*10*	*1.34*
*No Response*	*28*	*3.75*
*Indian*	*127*	*17*
*USA*	*496*	*66.6*

*Response Data for Question 3. Self-reported Gender Identity.*

**Table:** 

***Gender***	***Percentage of respondents (%)***
*Female*	*52.9*
*Male*	*46.8*
*Trans-*	*0.3*

*Response Data for Question 4.* On a 1-to-10 scale (10 highest concern), how concerned are you about the topic of ‘global warming.

**Table:** 

***Reported concern***	***Percentage of respondents (%)***
*1*	*3.8*
*2*	*2.3*
*3*	*4.3*
*4*	*3.2*
*5*	*5.8*
*6*	*8.0*
*7*	*11.4*
*8*	*21.4*
*9*	*15.3*
*10*	*24.5*

*Response Data for Question 5. Do you believe that substances commonly referred to as ‘greenhouse gases’ (such as carbon dioxide or methane) have the ability to warm Earth's atmosphere?*

**Table:** 

***Selected response***	***Percentage of respondents (%)***
*YES*	*92.7*
*NO*	*7.3*

*Response Data for Question 6. Complete the following statement based on your opinion. If Earth's atmosphere had no carbon dioxide or water vapor present within it, I feel that Earth's average temperature would be _________________.*

**Table:** 

***Selected response***	***Percentage of respondents (%)***
*About the same*	*8.8*
*> 10 °C warmer*	*20.7*
*> 10 °C cooler*	*29.3*
*5–10 °C cooler*	*28.4*
*5–10 C warmer*	*12.8*

*Response Data for Question 7. Based on your experience, through what means or mechanism do 'greenhouse gases' (such as carbon dioxide or methane) affect climate? (select all that apply)*

**Table:** 

***Selected response***	***Percentage of respondents (%)***
*By direct absorption of sunlight / moonlight*	*27.0*
*By absorption of infrared light emitted by the Earth and Earth's atmosphere*	*39.7*
*By destroying stratospheric ozone*	*52.8*
*By contributing to urban smog formation*	*25.5*

*Response Data for Question 8. On a sliding scale, how frequently have you encountered print or news media reports describing greenhouse gases effect on climate? (value of 10 is highest)*

**Table:** 

***Selected response***	***Percentage of respondents (%)***
*1*	*1.5*
*2*	*5*
*3*	*9.3*
*4*	*8.9*
*5*	*10.7*
*6*	*16.3*
*7*	*15*
*8*	*17.1*
*9*	*7.9*
*10*	*8.3*

*Response Data for Question 9. Do you attribute the phenomenon commonly called ‘global warming’ to human influence?*

**Table:** 

***Selected response***	***Percentage of respondents (%)***
*Yes*	*75.3*
*No*	*10.2*
*Not Certain*	*14.5*

*Response Data for Question 10. With respect to historical temperature data, scientists have recently commented that “Of the 17 hottest years on record, 16 have now occurred since 2000.” Given your experience, what factor(s) are primarily responsible for this observation? Feel free to check all that apply.*

**Table:** 

***Selected response***	***Percentage of respondents (%)***
*Solar variability – the sun is increasing its energy output*	*25.2*
*The statement in the question is not true – no warming is occurring*	*10.3*
*Oscillations in the Earth's orbit around the sun (Milankovitch cycles)*	*15.9*
*Increasing water vapor / humidity in the Earth's atmosphere*	*28.4*
*Fewer clouds are present*	*10.7*
*Human emission of greenhouse gases*	*70.6*
*Earth's surface is becoming less reflective (Albedo effect)*	*19.1*
*Shifts in ocean currents*	*25.5*
*other*	*13.2*

*Response Data for Question 11. Do you believe that aerosols (tiny micrometer particles of solids or liquids dispersed in the atmosphere) can affect climate?*

**Table:** 

***Selected response***	***Percentage of respondents (%)***
*Yes*	*66.7*
*No*	*8.9*
*Not Certain*	*24.4*

*Response Data for Question 12. Do you believe that 'soot' or ‘black carbon’ aerosol produced during combustion can influence atmospheric temperature and climate?*

**Table:** 

***Selected response***	***Percentage of respondents (%)***
*Yes*	*74.0*
*No*	*7.9*
*Not Certain*	*18.1*

*Response Data for Question 13. Based on your experience, through what means can aerosols affect climate? (select all that apply)*

**Table:** 

***Selected response***	***Percentage of respondents (%)***
*By scattering sunlight into space (albedo effect)*	*28.0*
*By absorption of sunlight*	*33.5*
*By altering the occurrence and characteristics of clouds*	*29.5*
*None of the above*	*19.6*
*Other mechanism*	*23.1*

*Response Data for Question 14. On a sliding scale, how frequently have you encountered print or news media reports describing the atmospheric aerosols effect on climate?*

**Table:** 

***Selected response***	***Percentage of respondents (%)***
*1*	*16.9*
*2*	*18.8*
*3*	*13.4*
*4*	*10.9*
*5*	*8.9*
*6*	*9.0*
*7*	*9.1*
*8*	*7.6*
*9*	*2.5*
*10*	*3.0*

*Response Data for Question 15. Regarding the topic of global climate change, which of the following sources do you feel has provided the majority of information used to generate your opinions?*

**Table:** 

***Selected response***	***Percentage of respondents (%)***
*News media*	*23.6*
*K-12 schooling*	*6.8*
*University education*	*13.0*
*Social media*	*11.5*
*Scientific journals or pop. science magazines*	*15.4*
*The internet*	*25.6*
*other*	*4.1*

*Response Data for Question 16. Geo-engineering temperature has been suggested as a means to lower Earth's temperature (if required). In this approach, aerosols (or aerosol producing gases) are purposely added to the atmosphere to reflect sunlight into space. Can you support this approach?*

**Table:** 

***Selected response***	***Percentage of respondents (%)***
*YES*	*48.6*
*NO*	*22.3*
*Not Certain*	*29.1*

*Response Data for Question 17. If you can support geo-engineering under certain circumstances, what global mean temperature change should be encountered before implementing geo-engineering of temperature?*

**Table:** 

***Selected response***	***Percentage of respondents (%)***
*Less than 1 °C*	*10.4*
*Between 1 and 3 °C*	*33.7*
*Between 3 and 5 °C*	*21.0*
*> 5 °C*	*11.7*
*Under no circumstances should geo-engineering be attempted*	*23.2*

*Response Data for Question 18. Are you aware that the eruption of Mt. Pinatubo in the Philippines in 1991 is believed to be responsible for approx. 0.5° cooling of the Earth during the following two years due to sulfate aerosols reflecting sunlight?*

**Table:** 

***Selected response***	***Percentage of respondents (%)***
*YES*	*30.2*
*NO*	*69.8*

*Response Data for Question 19. Which of the following do you feel is the most significant contribution to society made by atmospheric scientists?*

**Table:** 

***Selected response***	***Percentage of respondents (%)***
*Developing accurate weather predictions*	*19.6*
*Developing understanding of, and remediation of the ozone hole through control of chlorofluorocarbons*	*26.5*
*Developing understanding of, and reduction of urban smog by > 5-fold in many urban centers*	*24.0*
*Reduction of acid-rain / precipitation through understanding atmospheric chemistry*	*9.4*
*Developing models to predict Earth's long term climate*	*20.5*

*Response Data for Question 20. Based on your experiences, what is the most confusing or troubling aspect related to the 'global warming' phenomenon? Please explain in the box below (free response).*

*NOTE: A List of Responses are provided here as data. Many minor typographical or spelling errors have been corrected, however only minimal changes have been made to reflect the spirit of the respondent feelings. The responses presented here may be sentence fragments or otherwise grammatically incorrect.*

**Table:** 

Solar radiation passes through the atmosphere to the surface of Earth, where it is absorbed and then radiated upward as heat. Gases in Earth's atmosphere absorb about 90 percent of this heat and radiate it back to the surface. Human-caused global warming occurs when human activity introduces too much of certain types of gas into the atmosphere. More of this gas equals more warming.
No idea
Like this winter we had the coldest February reported since beginning. This is confusing because in general temperatures go up and then we have this extreme cold periods. But people should see the whole planet where temperatures go up and not local events like this February.
Ice melting (in) Antarctica and how it will affect coastal cities
The most troubling aspect is that even in 2017 a significant number of people believe that global warming is a hoax. If this continues within developed countries like USA, my county Bangladesh, will be the first to suffer the consequences.
Greenhouse gases. The media fails to explain what they are.
Lack of proofs
Galactic plane phenomenon and Solar activity
The most troubling aspect of global warming is the rise in sea levels that could result from melting of the ice caps because this will endanger costal areas and small islands which are inhabited by many of world's populations.
The fact that so many people believe it's not happening.
It is a natural cycle too
For me, the most troubling aspect is Fossil oil extraction because there's a lot of money and interest (for the money) in keeping that industry
the specific confirmed main reason for global warning and the specific main solution to the problems cause by global warming
I think its the fast melting of glaciers from the poles since it directly affects the rise in sea level and would lead for the low-lying islands to slowly sink in the future
Troubling: that governments don't always rely on scientific community to develop their environmental (or lack thereof) policies.
The most confusing is that temperature change has happened for the last 4 billion years and at some points even more drastic than now.
Sea level will rise and major cities across the world will become flooded, displacing millions/billions of people
Think it is all made up the earth will change. But people need to control greenhouse gasses and other harmful gases for our own health.
The fact that there are many contradictory views on its causes and possible management.
that many people are ignorant and do not seem to think we are being adversely affected by this phenomenon and that future generations will have a much harder time reversing its effects if we don't make significant changes to our lifestyles. engineers can solve many problems, and it's comforting that they have taken it upon themselves to engineer a solution to this particular problem, but engineers can't make people think differently, not ethically at least, and unless people realize and accept the magnitude of this problem, there will always be something impeding the progress which is trying to be made.
Humans
Not sure if human's cause it or if it is cyclical in nature. I am sure Human's contribute, but by how large a scale is the question.
The most troubling aspect is that small changes in temperature can affect ecosystems, and the increased production in carbon dioxide can cause detrimental pH changes as well. Example includes coral bleaching.
Nothing
I would say what are the causes of it and how can this problem if it exists be properly mediated when the world is so distinct and it would take a full on uniform approach to it.
How it can be prevented
Whether the warming trend exceeds normal climatic variations
Climate change doesn’t merely increase the overall likelihood of heat waves, say, or the volume of rainfall it also changes the flow of weather itself. By altering massive planet-scale air patterns like a jet stream
What are the drawbacks on this global warming and how it can be prevented.
None
we just destroy in just 10 years, now try to recover it in 100 years, what the funny
How the world is going to take measures and control the global warming.
GLOBAL WARMING IS A CLEAR AND PRESENT DANGER. STILL EVERYBODY, INCLUDING ME, TENDS TO BELIEVE THAT IT IS NOT GOING TO AFFECT US INDIVIDUALLY. I THINK WE HAVE TO THINK ABOUT OUR KIDS AND THEIR KIDS AND ACT ACCORDINGLY, SO THAT THEY ALSO CAN LIVE ON THIS PLANET
The use of plastics
GOOD SUBJECT
Global warming is happening at such a big scale that it's hard to predict on how to deal and eliminate it entirely.
BREAKING DOWN OF OZONE LAYER
global warming is national issue
global warming is very important in our atmosphere -in that case releasing some gases.
Green House gases, Absorbing the temperature and emit back to earth again, So temperature of the earth gets increased. By increasing green house gases, we shall get rise of temperature.
INCREASING TEMPERATURE CAUSES DROUGHT
Global warming is the gradual heating of Earth's surface, oceans and atmosphere. Scientists have documented the rise in average temperatures worldwide since the late 1800s. Earth's average temperature has risen by 1.4 °F (0.8 °C) over the past century,
Due to the large amount of consumption of carbon dioxide the rate of average heat on the earth increases this will adversely affect the lives on earth.
It is the known fact that Global Warming is the result of Human activities, but even after knowing very well, the Human race is not realizing the consequences of the future effect. One should try and understand the causes and try to control it rather than being in a confused state of what to do to stop global warming.
why the world countries are not serious about it
exact temperature changes
The impact on environment is badly affected
nothing to say
The major challenge is the awareness if these issues. Even being an engineering graduate, this concept is not clear to me that which other factors except pollution could cause greenhouse effect…
Its uncertainty of how it will behave in future and its consequences.
Global warming produces green house gases.
Steps to be taken to fight global warming, an exhaustive change in life and living.
Earth is only a very small spot on this vast infinite universe. How can a tiny object like earth can make such huge climate change on this vast infinite universal climate.
I think increasing temperature in atmosphere is the thing which affect tremendously.
The blame game resorted to by developed world on carbon emission by the developing world and justifying their own emissions, instead of finding lasting and equitable solution.
Global warming is mainly due to technology development many says and that is confusing to me.
Because of global warming mostly it will effect to climate
environment
POLLUTION IS MAIN ONE
MORE GROWTH IN AUTOMOBILE AND FACTORY EMMISION CAUSING GLOBAL WARMING
Scientists are terrible communicators. Their dry, analytical presentations of data fails to capture the imagination of the public. Scientists bury their findings in complicated graphs and jargon, and as a result the public often has no clue what they are talking about.
Global warming is taking place is reported but to prevent what steps to be taken and is not been taught and either implemented or forced to implement to save the earth. Why the authorities are seems to be lethargic in handling such a natural cause so lightly which may lead to great collapse of the universe.
It troubles me that the major industry players tend to overlook all the damage they are causing to the environment
Natural Destruction
No confusion, but we are the responsibility for our climate. Global warming is increasing day by day. We should take care our earth, so we should plant lot of plants and trees. We have responsibility to create and safe the green earth. Don't consume lot of dirty and pollution. Thank you.
pollution in urban city
tree
How to reverse the effects
If global warming is harming to our earth atmosphere, why countries not taking effective action to stop/decrease the same.
None
Deforestation is the major one in my view
Global warming communication should be passed to the people to aware the consequences.
Mentality of the human on global warming
nothing is there
Global warming and climate change are terms for the observed century-scale rise in the Ocean temperatures increase more slowly than land temperatures because of CO2 emissions are continuing to rise due to the burning of fossil fuels and … The popular media and the public often confuse global warming with ozone
Destruction of environment and air
geo-engineering
Acknowledgement of one's responsibility in this cause.
global warming is very harm our next generation people
More confusing about sulfate aerosols reflecting sunlight would effect the global warming.
Aerosols, never heard about that and could not understand.
altering the occurrence and characteristics of clouds
AEROSOL PRODUCING GASES
Global warming, the phenomenon of increasing average air temperatures near the surface of Earth over the past one to two centuries. Climate scientists have since the mid-20th century gathered detailed observations of various weather phenomena (such as temperatures, precipitation, and storms) and of related influences on climate (such as ocean currents and the atmosphere's chemical
It is very sad that temperature is rising yearly. It is hard for the scientist to find out the reason of temperature rise.
support for climate change
Greenhouse gases are adding up to global warming now because it was there since 80's.
global warming is very danger
CARBON MONOXIDE IS MOST HARMFUL FOR OZONE LAYER.SO THE EARTH TEMPERATURE INCREASED.
Countries should spend more on research on Global Warming. Media should put it in such a way to be understandable by a common man.
Global warming and climate change are terms for the observed century-scale rise in the average temperature of the Earth's climate system and its related effects.
How can we prevent 'global warming' due to natural aspects.
that we (are)not doing enough
How is it possible to develop without affecting the climate
how it increases and decreases
Average temperature of earth is rising causing global warming
Why the countries are not taking necessary steps towards it and informing it since antiquity
Ozone
Global warming and climate change are terms for the observed century-scale rise in the average temperature of the Earth's climate system and its related effects.Multiple lines of scientific evidence show that the climate system is warming.
Its clear
inaccurate data
Politicians rejecting science
It's not clear if certain policies against global warming could have unintended economic consequences
Tons of opinions by unqualified people with no specifics, just because someone said so is an empty glass.
I do not understand how there can be people that do not accept the science that prove it.
To what extent is global warming caused by man?
Gaps between scientists and the public…
ozone on earth
The morality of asking under developed nations to not do the exact same thing developed nations did to enter into the first world.
Heat wave, rising of sealevel and the shortage of rainfall
cutting trees
The term global warming itself. How "global temperature" is defined
There are claims and counter claims in as to whether there is "global warming" or not.
We can't be sure if it's really happening or not
Troubling aspect is a possible tipping point.
TERM ABOUT LOWER TEMPERATURE OF EARTH
The confusion based on the opinions of different scientists regarding what is causing 'global warming' and the melting of the ice caps. However at the same time it has been stated that the Antarctic has increased ice formations.
we know that we are the problem but we do nothing to change our life style FAST!
There are conflicting accounts of how global warming come about and hearsay are distracting people from solving the problem
Paying for CO2 emissions
The lack of local estimates of the effects of the warming.
failure to disconnect financial aspects from science
I find it amazing that there is such a consensus in the scientific community.
I cannot comprehend why people are against the facts about the climate change. Why would scientists lie about something like this? Like… From France to Japan to the USA to Russia. They are all lying for the same conspiracy theory? People are stupid and politicians need to start listening to experts on this topic to take action. Otherwise, the "slight" increase in the mean temperature will start having devastating effects on the biosphere and a lot of species will die because some idiot playing video games for 15 h a day doesn't believe in modern science and disperse false information about his baseless claims on the internet sphere.
The effect the petro-dollar has on negotiations and why they are stalled.
First of all, I think that pure nature has determined it, and if that is not the case, then it is definitely new technology, radiation, folly of the people ….
the troubling aspect related to the global warming phenomenon is that we know what is harming our earth and the majority "uneducated people" choose not to do anything about it because they think "it wont happen in their life time" or they just dont know. but its not our life we are trying to protect its the future!
The lack of moisture/water in viable areas will contribute to the destruction of the earth
I am not as well read on the subject as I would like to be. Perhaps the most troubling aspect of this topic is that there are many people with little to no knowledge on the subject that have formed very strong opinions either for or against the idea of climate change. What is even more troubling is that these people may even serve as a source of information for many other uninformed people.
The reason why it is so disputed
The effect the humanity has on global warming, and to what extent warming is a natural occurrence
The rising of our oceans and ocean acidification
People in political control of funds to make corrective changes are more interested in protecting financial interests of big business than the environment.
The scientific community has reached a 97% consensus that climate change is influenced by humans, yet many people still doubt that this is true.
getting an agreement that it even exits, to many different versions of what it is and what causes it
That debate is not allowed, that models that have consistently overpredicted warming are being used to decide policy, and anyone who expresses any sort of doubt is labeled a "denier" on par with a Holocaust denier. The arrogance of scientists astounds me- they're so sure they're right, that their (obviously flawed) models are correct that they don't want debate. We've stopped the question at "is the earth warming" when there are so many more- how much is caused by man (it's certainly not 100%), what can we do about it? If we do something, will the benefits be worth the cost? Is it better to pay to adapt or pay to try and change the climate? Who will most be harmed by reducing emissions and how can we ensure the poorest of the world aren't paying?It's so cruel- we in the West benefited from abundant and cheap fossil fuels and want to forbid the poorest of the world from the same benefits we had.
News media spreading false conclusions of "scientific consensus when no such thing is true. Respected scientists disagree with the so-called "consensus". Over reaching hyperbole from the EPA and previous presidential administrations.
I don't understand why people do not recognize that the planet is dynamic and ever changing. Yes, mankind is effecting the current rate of warming, yes rising oceans will displace millions of people and current low lying cities and countries. The earth will adapt because that is what is has always been doing, changing and adapting.
I recall reading quotes from climate scientists that suggest that greenhouse gas levels are approaching or have passed the tipping point for global climate change.
Sea temperature rises and increased absorption of CO2, disappearing glaciers, glacier holes and melting ice caps. Sea-level rises will create world-wide waves of climate-change refugees.
Diametrically opposing articles on similar subjects.
Anti-science propaganda by right-wing politicians and media.
Political Bias
That people refuse to believe in it
removal of vegetation prohibits carbon dioxide from being absorbed by vegetation and converting to oxygen
Disinformation - "fake news" - spread by fossil fuel spokesmen like Donald Trump and Scott Pruitt - claiming that climate change is not real, a hoax, is not happening, is not caused by humans. Worst are claims that there is not actually scientific consensus that climate change is happening and humans are primarily responsible.
Politicians who are ignorant of, don't care about or value science, or who have vested interests in doing so, making false statements related to global warming and climate change.
When regions become colder in connection to global warming.
How small of a change in mean world temperature relates to such catastrophic physical changes in the planet.
All of the hyperbole around the sky-is-falling attitudes. Everyone said we were going to nuclear war in the 50s–70s yet here we are. Not enough science to back up any of the claims.
I dont understand why there was a ozone layer depletion in humanless place
convincing other people that we need to do something.
What exactly is causing it
the fact that scientists' 'expert' opinions are all so diverse….
no real scientific data to prove yes or no
how it trickles down to everything
Conflicting information perpetrated through fake data from deniers of climate change.
Whether the effect is overstated or not.
That it is something that people still believe is not happening. Seriously worries me.
why people wont listen
how it seems hopeless to fix
How to prevent the phenomenon and the way to deal with.
How to stop it
The conflicting news media and scientific research, makes it hard to know what to believe
No one knows how to talk about it. We still don't know a lot after so many years.
That it has nothing to do with the planet. But it is complete propaganda wrapped up in political agendas and movements. Esp. the globalist movement of creating a global problem that needs oversight from globalist themselves.
The intrusion of politics and the potential for deception from all sides.
How easily people are swayed by puns and name calling to observe anything else.
The most troubling aspect is the government doesn't care because it doesn't pay them to care.
It is just hard to KNOW what exactly is causing the fluctuations. I firmly believe the Earth does have cycles that heat and cool, but I could also see how we may be impacting that to a greater extent with the things we do.
that scientists have altered data to show an increase in temperature
Whether it is true or not…I haven't seen any definitive research.
People not believing it occurs
The presumption that humans are responsible for climate change. If you believe history, the earth has experienced many warming and cooling cycles. These occurred well before man's theoretical influence.
Lack of interest in development of better technology.
The confusion about the true cause. Is it man caused phenomenon or is it naturally occurring
the percentage impact by humans
People who do not care about the environment, but only profits or selfish pursuits.
Nothing, I think some people are just Dumb!
the accuracy of its models
its a very complicated subject and there really isn't a way to reverse the problem.
IS THERE REALLY SCIENTIFIC PROOF FOR CLIMATE CHANGE.
The most troubling aspect is that some of those that are in power do not believe in global warming.
Not all countries have the same feelings towards global warming. For example, one country may implement a solution that helps slow global warming, but other countries may not follow. If a very large country, such as China, does not do anything to combat global warming, the rest of the world has to suffer because of them.
Carbon dioxide
Whether it's happened to this extent before.
Inconsistency in scientific data
The earth goes through cycles, it heats, it cools. When it reaches a point it will eventually cause the earth to cool again. Spending billions of dollars to drop the worlds temp. by a half a degree is a waste of money
All the different conflicting sources of information in the media.
some people dont believe
Everything in questions 1–19 - not easy to explain to those without scientific background
THE FACT THAT THERE IS NOT A TRUE 'CONSENSUS' OF SCIENTISTS ON THIS MATTER
The long term natural temperature cycles which the earth goes through without humans being present.
It's a complicated topic and the results of changes take years or decades and are not instant. It's hard to know what is working as this is a long term problem, but we as a society are focused on short term problems.
It is so complex the 'average citizen' can't wrap their brain around it. Faced with what we can't understand, we simply stop listening.
How much other factors contribute to global warming and how much of our knowledge is based on data collected in the "recent" past? I feel like much of the public isn't looking more than 50 years into the future.
The fact that most of the models that prove global warming have data that is made up and later filled in.This is the reason that the models are constantly adjusted. Only real data should be used in the models.
Too many varying sources on the subject.
The most troubling aspect is not about climate change itself; rather, it is convincing people that climate change exists. I find that to be perplexing.
Always mentioned it is human caused, but never addressed is the overpopulating of the earth by humans and controlling that as a necessity in the global warming equation.
That it has become a political issue and non-scientists weigh in on the argument.
The earth used to be much hotter with no ice at the poles at all.
Multiple sources having multiple interpretations of global warming- supported with valid information.
Religion debating global warming merits, in order to keep the scare money rolling into church coffers.
there is little correlation to the extreme effects to our daily lives, so it almost makes me not care or not want to try to do anything. It needs to be presented as a tangible problem that we can each help solve instead of a lost cause
The flat out refusal to talk about all possible aspects of climate change
certain segments of societies outright denial of it
Unfortunately, the most confusing and troubling aspect is the inability to find conclusive or near conclusive arguments that would allow human-kind to effort together to solve the issue. We mire in disagreements as we watch the future of our people, as well as every other species fade.
The drastic changes in weather
The most troubling aspect related to the 'global warming' phenomenon is that it is affecting the plants and trees, the oceans and rivers, and the atmosphere.
The earth has had historical climate changes.
The aerosol bit, I wasn't aware that existed. Also, unrelated to global warming, please change your gender question. As a transman, I had a very hard time choosing between trans- and male. A better way to do this would be have a male option, female option, non-binary option, and then a fourth option that would allow the participant to write down what their gender is if any of the other three options didn't fit.
The fact that many people don't believe in climate change.
that i dont really give a sh_t. Im fine if humanity kills itself off
The troubling aspect is the hate both sides of issue have towards one another.
Not sure
not sure how true it is
We still need to use fossil fuels so we don't have a complete and absolute method to cease our civilization's ability to destroy the environment.
It is hard for me to believe that adequate testing was even possible to see the effects that are taking place against the ozone.
Earth goes through warming and cooling cycles naturally. It is rarely mentioned and does not seem incorporated into any study.
Over 90% of scientist worldwide say global warming is cause or increase by man's activity, but many people still do not believe it.
How people can argue it is not happening. The only thing that can be argued is whether it is due to human influence.
That it affects us in ways we can't concretely see in short spans of time.
The most troubling aspect is the constant disagreement and downplaying of global warming by some scientists but overwhelming by politicians and industry.
That would be the fact that some people don't believe Global Warming exists.
The most troubling thing is that people who know its a problem don't care enough to try to fix the problem.
That it's supposedly warming, but my winters are getting colder and summers getting cooler.
albedo effect
can it be reversed
That people keep disagreeing on if its happening or not.
The lack of data. If the world has been in existence for many billions of years and we only have about 150 years worth of data how can we truly know what the weather patterns should be.
The uncertainty of predictions of temperature change over long terms.
There is a ton of information out there about it. A lot of it is conflicting depending on which news outlet is releasing it. It makes it confusing for a lay person to really understand the issue.
Highly politicized misinformation everywhere and the fact that we plan to curtail economic growth through dubious means such as cap and trade. I feel the market is more than capable of responding to any threats from climate change and we should let our technology continue to develop so we can counter any ill effects we come across.
What confuses me is how people can deny the fact that whether or not the earth is warming, it is definitely changing. And it is being killed by all our waste regardless of climate.
CO2 pollution
That some still deny it.
How far ahead you have to plan to make changes. We could change for the better now but the effects may not become known for years down the road.
Weather is becoming warmer. It's very harmful for us and for me also. I am very worry about it.
We really dont have an idea
I think the most troubling aspect besides the warming of the planet is the rising sea levels and melting icecaps that may cause a lot of places submerge completely.
The harsh debate--a simple understanding would weigh far more than any childish dilemma.
everybody needs cars to get around so how can we reduce emissions without sacrificing our livelihoods
Current statements are only looking at a VERY small amount of data. Studies of long-term data indicate that warming/cooling cycles are very common in earth's history. I'm concerned that by taking a short-term view "we" may cause more harm than benefit.
The fact that celebrities and high ranking officials who support it are acting in a way that goes against their beliefs… ie multiple homes, private jets, etc. They aren't helping their cause by acting like hypocrites.
that people don't believe it exists and campaign against.
The prevalence of disbelief and denial regarding global warming among certain members of the general public.
The attention to the problem now that it effects us more then in the past.
all of it.
population will always increase, so i believe global warming will as well
UNDERSTANDING AND EDUCATING THE PUBLIC
most troubling aspect is ignorance by policy makers
Because there are so many brilliant people an some say its one way and others say its another and we really have no way of truely knowing
Its hard to tell if it is in fact caused by humans or if it is just the fluctuating climate that the earth has experienced for millions of years.
That people don't believe it.
Differentiating it from natural temperature fluctuations that have occurred in the past.
It is difficult to know what is fact and what is political. Plants need carbon dioxide to survive. So, now CO2 is a poison responsible for warming the planet to the point that human survival is at risk? Also, the sea level was already supposed to have risen several feet (according to media from a decade ago). Sea levels seem to be the same to me.
The politicizing of facts. People disregard science because they don't agree with it.
all the doomsday scare-tactics that science and media are currently using
The amount of contradicting information produced by different media outlets
The lack of data from the first 4.5 billion years of earths history
I only have limited knowledge in science
effect of everyday actions
none
How there is more politics and insufficient actual science in discussions of global warming and climate change. Too many assumptions, and not enough actual science.
I don't understand why we don't use more nuclear power.
denial by our government
Migration patterns.
the most troubling aspect of it all is that not everyone is doing all they can to reduce their carbon footprint to reduce global warming
All countries view global warming differently. There is no one solution.
Nothing about global warming is confusing or troubling to me. We have nothing to worry about.
It is troubling that we do not have enough data to perfectly predict the changes that will occur not only by our current human behavior but also any behavior we might exhibit to counteract global warming and climate change.
I fear that it will take collective action from every major nation, making it difficult to solve
Viruses and diseases stored in ice for which we have minimal resistance to.
I think this a plan to increase government control over society.
There is too much politics involved in what is supposed to be a scientific endeavor. Too difficult to determine if data are unbiased.
I feel there isn't one.
It is how exactly global warming effects temperatures
What I find most troubling is how the whole thing has devolved into a political weapon and we're left arguing is very existence instead of how to mitigate the worst effects of it.
All the hustlers trying to make a buck off of whatever public opinion they feel they can leverage for the most money.
Other planets also warming up
it is far too politically motivated. All the media focuses on the political side, and not the science side. And even the science side is confused and cant come to an agreement on the reality of the situation.
There are too many opinions about it, and it is difficult to know which one to trust/believe.
How much it is caused by each factor.
Global Warming Denial
Making vehicle inspections over reaching and hard to pass for people with emissions lights on. It's a big fat money grab, a way to push already poor people further in to misery in the name of "climate change"Like George Carlin said, we are arrogant to think we affect the billions year old planet. The earth will shake us off like a bad case of fleas when it is ready to.
The most troubling aspect is the lack of belief its most proud commenters have. Of course, there are those like the No Impact Man, who I am convinced by, but people like Al Gore, or even the average Greenpeace hippie celebrity shouting about climate change on social media- these who still use iphones, home internet, gasoline powered cars, aircraft, and boats are hypocrites and liars. We will never make environmental gains while still expecting our lives to remain the same.
That each side is to busy pushing an agenda for money that it is hard for average people to see the big picture. The whole solar system has been heating up, Mars is also having a " heat wave" the same period of time we have.
All of the research looks biased. There is obvious evidence for arguments going both ways, but the facts of the matter are that no one presents both sides of the facts and shows everything rolled up into one. I believe we do not know enough about the past weather changes and weather cycles to determine if humans are having any type of true negative effect on the Earth and its atmosphere with our chemicals and their byproducts.
The consistency with which the temperatures seem to rise.
No one can agree on anything about it.
Most troubling is how some people can deny science based on some idiotic political view.
the most troubling aspect to me is the fear that has been conjured about the topic. I'm not convinced one way or the other about global warming, and if it is in fact happening I think it could be completely natural, or that humans could've influenced it. There were obviously drastic climate change events in the earths history long before humans.
Combating misinformation.
Misinformation from conservative news media and groups.
that nobody seems to be taking the aerosol approach to reducing global temperatures seriously - I have seen very little mention of this in the news
The flip-flopping of specific "well respected" scientists over a 30 year course.
The most troubling aspect is the continued collapse of ice shelves in Antarctica which may lead to rising global sea levels, endangering many people.
The aerosols
The fact that people hold themselves in such high regard, they think they can stop the Earth from doing what it has done by itself for billions of years.
I don't think there is a whole lot of evidence behind it.
Earth's temperature is going to change no matter what.
people are the main cause of global warming and part of that is the destruction of plant material to add more buildings and paved surfaces and removing trees and other plant material that absorb carbon dioxide and release oxygen for human use.
The avg Earth temperature fluctuates from year to year.
varying weather predictions
The effect of aerosols and geo-engineering
The fact that so many people, especially in America, still think it's just a left-wing political conspiracy with no evidentiary basis
The fact that so many people (especially in America) still think global warming is a left-wing conspiracy with no evidentiary support
The rise in ocean levels due to ice/glacier melting as a result of global warming, and why water displacement can't be controlled through man-induced evaporation.
The denial of man's role through use of selective data or misconstrued arguments
global warming misinformation spread by corporations to make people think global warming is not real.
I find hard to believe that political and economical interests are entangling this studies.
The most worrying thing is that we try to counteract it but it continues, I think we should do much more for our earth.
The amount of information that is contradictory, it is necessary to reach a consensus to work together in order to obtain the answer we need to create to transform our reality into a better one.
I think the way it's explained to the common public, using many technical terms or trying to explain it in ways people simply doesn't take it as a serious matter. When you see dead bodies due to a traffic accident, you take it seriously, you can see and almost feel the consequences of irresponsible driving, but regarding "global warming", people see the consequences, but they simply can't associate them to this problem.
There are too many interests at stake, international policy decisions are needed to address, not global warming, but the new ice age ahead.
The uncontrolled human contamination that to continue like this will destroy our planet
Despite of all the things are happening in the world, there are still humans who don't care about the environment or how to reduce global warming. In a few years earth won't be the same
Melting icebergs
Studies coming up with drastically different results and the lack of governance in the world
The fact some people deny it.
Well president Donald Trump doesn't even believe in it.But I didn't vote for him.
The fact that people do not care or think that its an issue. Its only going to get worse.
Despite global warming being widely recognised, human kind are still not playing a big enough part, to minimize this
Is it solely because if human activities?
Why is it so controversial? Some sources say it isn't happening.
What is exactly causing global warming
The awareness and significance of how the impact of each individual on the environment
Understanding how it occurs
Potential effects on inhabited areas and resources
CAN'T THINK OF ANY
The most confusing or troubling aspect related to the global warming is the effect of human activities which contribute to the effect of global warming such those of the factories.
How the U.S. President can essentially deny its existence
Im not sure
Understanding what all is contributing to global warming and learning how to prohibit those emissions.
ocean levels rising.
everything. Nothing is clear and there is too much noise surrounding the topic to know what it is really true.
over heat affected people
Water pollution is a global warming
refers to the recent pattern of temperature increases in the earth's atmosphere and oceans, attributed in part to human activity. The scientific evidence for global warming is overwhelming, but the political debate continues. Part of the reason for the continuing debate is that climate science is a complex subject. Climate itself is the result of the interaction among dozens of factors. Because of that, you can't just observe changes in one element and connect them to a specific climatic effect -- which makes explaining global warming a challenge.
Global warming, the phenomenon of increasing average air temperatures near the surface of Earth over the past one to two centuries. Climate scientists have since the mid-20th century gathered detailed observations of various weather phenomena (such as temperatures, precipitation, and storms) and of related influences on climate (such as ocean currents and the atmosphere's chemical composition). These data indicate that Earth's climate has changed over almost every conceivable timescale since the beginning of geologic time and that the influence of human activities since at least the beginning of the Industrial Revolution has been deeply woven into the very fabric of climate change.
Earth's surface is becoming less reflective
change in temperature and drought
Pollution is the main thing
Global warming are caused by we human beings only.
global warming leads to secure
which are the main causes for global warming and how to avoid it. More and more and more awareness should be created among people to avoid global warming. Nowadays itself we are suffering a lot due to warmest summer days. So many and immediate steps to be taken by Govt. of all the countries.
air pollution
acid rain i think the most powerful one
Global warming causing affect to human and nature even we all don't know what happens to our nature and us in survey I got confused about black carbon
How to save the earth.
WE HAVE TO TAKE EACH STEP AND RESBONSIBILITY TOWARDS REDUCE GLOBAL WARMING
good project
Humans has to respect the environment and do prevent global warming and also climate change is also on of the reason.
most dangerous thing on earth which no one take seriously
it was very dangerous
Although everyone is talking about global warming, no proper governmental measure is taken by any country all over the world. I could not find the reason behind it. I find global warming concept is fake.
None
Increasing temperature is the most troubling aspect.
Causes and differing ideas to mitigate it
The increasing carbon dioxide by various aspects such as air pollution, factory emissions
Global Warming is the increase of Earth's average surface temperature due to effect of greenhouse gases, such as carbon dioxide emissions from burning fossil fuels or from deforestation, which trap heat that would otherwise escape from Earth
the term about atmospheric scientists…
NICE
Various gases today are contributing to the global warming. i think we need to control the emissions or find effective substitutes for those harmful gases
THE MOST TROUBLING ASPECT IS THE REASON FOR GLOBAL WARMING.THE REASON IS ALMOST NOT FAMILAR TO COMMON PEOPLE
Majority say that global warming will lead to an inhospitable earth. The change in monsoon patterns in my state make me feel it could be true. But I do not see any Government feel that sustainable development may be a better approach than industrial development. It is a very helpless feeling! Can't those human beings who live a very luxurious life think that they are destroying the earth for their own descendants?
The pushback from those in denial.
I don't have enough knowledge about this matter.
The whole aspect is very confusing. I cannot answer this. You wish scientists would explain it in lay mans turns
Humans from the beginning of time breath out carbon dioxide so how and why is it now causing a problem?
The most confusing part is why is it so expensive to purchase items that can help save the environment
Is it true or not?
That there seems to be no alternative to slow down the process
Possible solutions as well as possible sources.
How to control it
The fact that the government wont do anything about it.
Differing opinions on what is causing global warming.
The main cause of global warming and how to deplete it.
The scientific process is often not explained in detail in school, leading many to be confused about it and also its validity.
I am not really sure as to how much of the global warming is contributed by the humans and how much if it is caused by nature itself.
GOOD
no one cares
Disruption of thermohaline circulation
The money behind it.
chem trails
Why people are so reluctant to acknowledge the problem or take any steps to help solve it.
How significant is human act related to global warming compared with natural cycle.
that we don't hear enough about it
what is the main cause
YOU DO NOT KNOW WHICH TYPE OF MEDIA TO BELIEVE
Spray from the can increase the heat in atmosphere
increased temperature trends
Preventative measures should be taken now and no one is doing anything.
Pollution from factories
The lack of consensus between scientists and political media. I usually expect testable data to speak for itself, but there seems to be much debate about global warming.
It is very difficult to determine what is fact and what is only opinion.
Whether small individual changes like driving hybrid cars can actually make any impact or whether the climate is going to warm anyway
The most troubling is that some people still do not get it.
How politicized it is - people who stand to profit off of fossilized fuels are deliberately sowing confusion and doubt in order to influence policy in the US, to the detriment of public and environmental health.
The use of scientific terms specific to atmospheric science with which I am not familiar and of which I have little understanding can be very confusing. I think this can cause some misunderstandings among people who are not able to grasp what scientists are talking about when they speak about climate change. It is easier for people to dismiss that which they do not understand and climate change is one of the things that most people do not understand.
The multifaceted cause of global warming. It seems as though news outlets try and simplify the topic but that can cause confusion when intaking other information on the topic.
Climate change deniers in high powered positions along with little scientist representation in Congress
idiots who won't listen to scientists
The fact that there are government officials that think global warming isn't real. They try to prevent research into the subject. They say that 97% of the worlds scientist are apart of a hoax to convince people that global warming is a thing.
Probably the mis-information the media puts out.
The most troubling aspect is that we have such limited data - we don't have hundreds of years of daily high and low temps across all parts of the globe on which to base our conclusions. So we are making broad assumptions based on limited information. I also find it troubling that whether we blame it on cows or aerosol sprays, our climate is changing and we want to protect our home. The world must be protected for future generations because this is the only home we have.
I think that just looking at history, we may have records of the temperatures in the last several years, but how do we know the temperatures from thousands of years ago and how can we positively say that since the year 2000 has been the highest temperatures? I guess I would just need to study the subject more to really thoroughly give my opinion.
Although I don't agree with the statement, I have heard many argue that because there is still snow we aren't really having a 'global warming crisis.'
False data being used and the length of recorded data versus the existence of the earth
weather prediction is still so far off the mark / temperatures keep getting higher
There seem to be many components that tie into global warming, yet not all of them are discussed. I feel that it is important to show all of the information, not just a sliver of it so that the population has a better idea of what causes global warming and how we can reverse the effects of it.
Climate Deniers or Consumer Apathy and the Political Implications of Island Nations Losing their Islands
The idea that humans are ignoring the affect we have on the earth.
The severity of the problem, and yet the lack of concern from people with political power especially in the federal government.
When uneducated people are so staunch in their unfounded opinions in the face of real facts generated by thousands of climate scientists who have dedicated their life's work to figuring this problem out.
The fact that some people still don't believe global warming is a problem is terrifying.
I think the most troubling aspect of the global warming phenomenon is that it is a very serious issue that most people don't take seriously because it happens very gradually, and they don't understand the impacts of global warming. I'll be honest, I don't really understand the ins and outs of global warming, but, from what I understand, it effects our resources. I can be wrong, but I'm pretty sure desertification is either caused or exacerbated by global warming and this negatively impacts the ability to grow crops. And, whenever there is limited resources, people will be willing to fight or go to war over them if need be.
I am troubled that so many people do not realize the urgency of making lifestyle and business changes to prevent and reverse global warming.
I am not familiar with aerosols or I cannot remember previous lectures over the factor.
The percentage of scientist that people often quote that believe in global warming seems to be an odd sample to pull from.
How much is human caused and what we can do about it.
The lack of belief in the presence of evidence.
Carbon tax
It seems like scientists do not look at the earth's climate over a very long extended period of time. Also, the concept of the conservation of energy states that nothing small can change the temperature of an entire reservoir such as earth's atmosphere.
The surface will get more heat
Global warming is being further fueled by geoengineering methods that have already been in place for several decades. Geoengineering is not a proposal that is being considered. It is a current practice that is causing severe climate damage and the threat of global extinction.
The weather pattern is off with the month.
There is just so much rhetoric on the subject by politicians using the topic as a means to an end. They use scientists who are nothing more than political puppets to support their claims. The scientific community needs to be reaching voters who believe what the media especially the quasi-media tells them.
It is troubling that the climate is becoming more unpredictable and that the topography is being affected.
Whether it is true or not needs to be stated so even the dumbest person can understand it.
The actual process and what is occurring
the fact that some people still refuse to believe in science
to be honest, all of it.
what we can do.
There are too many conflicting reports and inconsistencies. During the last weekend of April, 2007 North Carolina was experiencing record highs while The Plains and Midwest were under Blizzard conditions. In March of 2017, Pennsylvania experienced one of the worst storms they have had in the past 10 years. How is that 'global warming'?
The fact that it is always being stated that climate change is not true. This is troubling because it is happening.
There is a lot of conflicting information and it is unknown how much is caused by humans and how much is environmental.
I am troubled by what the long-term consequences of our inaction will be.
We have no real answers as to have to stop it
CONFUSING? NOTHIING IS CONFUSING IT IS VERY CLEAR THAT WE ALL HAVE THE PART OF MAKING IT WORSE
That is the greenhouse effect. I still do not understand the mechanism because the polar meteor is not melting with the current temperature
Reducing smog in big cities
How people can think climate change is a partisan issue is the most confusing part of the whole thing.
The media portraying it as a hoax is troubling.
I am unsure
There's so much information with global warming that I never feel I know enough to form a real opinion.
Prior to this, I was not aware of aerosols and would like to learn more about it because after taking this survey, it seems quite confusing.
Global warming is very confusion because there are so many different viewpoint on it. Some scientists say it doesn't exists and some say it is a huge issue.
Facts
the fact that there continue to be flat earthers. Why is it that people in power do not believe science even when it is hitting them over the head!
most confusing is the multiplicity of ways it occurs
The Global Warming Aspects and Polar Ice Caps Melting.
People talk about it but to get people to actually change their behavior to help reduce human contribution to global warming is very difficult to accomplish.
earths atmosphere seems to be hotter than years past
whether it is caused by humans or if it is a natural process
why some people don't believe in it
Many Pros & Cons … from different sources on the issue
That the Earth has gone through periods of cooling and warming for millions of years.
Rising sea levels will cause mass disruption in population settlements. This will probably cause major conflicts to erupt as people seek out new places to live.
Company using coals
All the different things that can be contributing to it. Its also not talked about enough for people like me to completely understand whats going on
The sea level is rising due to global warming.
That we need more information
I'm deeply troubled by the rate at which humans are consuming resources. Our resources are not infinite.Clear cutting rain forests for more paper….trading lungs for trash….creating a need for more landfills. And on and on it goes
It is confusing regarding if is really happening. There are too many conflicting viewpoints even though there is proof it is happening.
That so many people don't seem to realize that the Earth goes through cycles of varying climates. You can't just say that humans are the cause of global warming - contributors, probably, but that's most likely not all that's going on.
I don't know much about it
Not having enough evidence
How to stop it
All of the technical terms used to explain the issues.
Figuring out how to stop it effectively
How many people don't believe it's humans at fault for this problem, only living beings need to worry about the state of the environment, the planet earth will keep spinning whether it has life or not. We need to care or life on earth will not be sustainable.
Trying to ascertain which answer would be the best choice to combat this issue.
How we can change what we are doing to reverse it
There is so much conflicting information that it is difficult to wade through fact and fiction
How much human contribution affects global warming.
none
people that make you feel stupid for believing it
The most troubling aspect is that people are confusing science with politics. Science is science. Facts are facts. It is a FACT that climate change is real. It is a FACT that humans have contributed to the warming of the Earth. It's disturbing that people know the truth but lie for their own personal gain.
I am most confused by accredited scientists on the subject releasing opinions that drastically oppose each other in terms of the causes of global warming or if it is even occurring.
Everything was very clear!!!
Humans not properly taking care of their trash.
It seems like no 2 people have the same idea of how to solve the problem. Many feel that there is a problem but the blame game goes on and on.
The relatively few number of years that humans have been tracking the Earth's temperature, weather, and climate leaves room for doubt. The Earth is very old and based on our limited data, I am unconvinced that we fully understand changes in the Earth's climate and can attribute the causes with accuracy.
Hard to find non-biased sources for the general public. public opinion heavily swayed by opinionated news media and social media.
Certain arenas have made the term politicized and therefore certain populations won't believe the ample science.
God controls it all and many do not believe/understand
total carbon output and reduction in carbon filtering mechanisms
Multiple contributors to global warming and how each contributor actuates global warming.
That a large number of people either don't "believe" in it or refuse to accept it because it would require altering certain aspects of their lifestyles/sources of income.
Conflicting information and heated debates that do not address the same issues.
It's most troubling to me how so many people still continue to deny its existence.
A troubling aspect is how scientists can't come together more on the subject.
some of this is confusing
That some idiots don't believe it existed
Don't know if it's really happening. Yes, it's hot; but it's been that way before.
I honestly don't know that much about specifics, but I know that scientists at several agencies are concerned and have documented changes in temperature, weather patterns, and melting of ice. I wish I had time to learn more of the details, but I trust those scientific concerns. I'm not sure why people make such a big deal about whether humans are the direct cause or not - we are playing a role (if not the sole driver) in change, so why not do things to slow down the progression?
not sure
the humidity and high temperatures we have been having
Honestly, everything about global warming is confusing to me and a little hard to comprehend.
The earth is getting warmer and there is nothing we can do about it because people won't listen
WHAT ARE THE LONG TERM EFFECTS OF GLOBAL WARMING IF NOTHING IS DONE ABOUT IT?
That much of the global warming is caused by humans.
Usually warm weather
Extreme Weather
how we can combat it
Lack of education!
We only have data for such a short period of time, the earth has been alive for so long it's hard to say our data is meaningful.
What is actually the true cause, and will we ever know?
That people do not believe it.
Not sure if this is a true phenomenon or not
Too much differing data out there and dishonest and uninformed people claiming to be experts.
why is this happening now
The changes in the ocean levels
That they don't push the issue enough about whats going on and how it should be fix. They let it go until something major happens.
It seems to be the inevitable. I don't believe anything can predicted because the world has changed drastically in the just the past ten years.
Why no one can agree on whether or not its happening
Anything scientific! Like when you talk about remediation of the ozone I don't know what that word means.
Not being able to prove the prediction of a long term change in the Earth's climate
misinformation in the media
why on the east coast is it getting cooler than?
Why it's occurring
People who are complaining about leaving a carbon footprint that is affecting global warming are the people who are contributing to the global warming issue like everyone else, but pointing fingers at others, and trying to be the heroes.
I wonder if we can control it at all. This earth has gone through global ice and heat for millions and millions of years, and humans had nothing to do with it.A part of me believes that this earth will do whatever it wants. We have only been keeping records for what the last 150 years? out of the billions that this universe has been around
Social media and other venues spreading false information which sadly most people believe. The study and reading of educational journals and papers are not common practice. We are like sheep, a group mentality. Information is being force fed to us and we believe it. There is also not enough people that care.
well not sure about confusing but troubling is the melting of polar ice caps
That a non-zero percentage of people think it isn't real or caused by humans, or not a threat.
no one can seem to agree on it.
I remember in the mid 1970s these same scientists went on TV claiming we were headed for a second ice age. They are full of shit.
Rising sea levels.
none
Everything! There are so many factors, so it makes it hard to know what to target.
need be
The resultant rise in sea levels from melting of ice floes and glaciers.
What seems to be most confusing is the variety of proposed causes. There is no question that there is global warming, but scientists and politicians argue over the cause(s.)
All of it
There is a lot of information about global warming that is explained in scientific jargon, which can make it difficult for a layperson, so to speak, as myself to fully appreciate the scientific elements involved and the complexity of this topic.
I AM CONFUSED BY THE MULTITUDE OF SUGGESTIONS ON HOW TO HELP SOLVE THE PROBLEM. I DON'T KNOW WHAT WOULD WORK OR WHAT WOULD HURT.
That many believe it to be a myth, and thus do not wish to do anything about it.
That humans are at fault
TOO MANY PEOPLE WITH TOO MANY EXCUSES AND REASONS
I believe people are twisting the facts to make them agree with their thoughts and agenda.
It is affecting the earth's atmosphere
I don't understand a lot of the things in these questions, and I don't think a lot of people would either. Explaining why is the most difficult part, especially since it is probably a combination of many things. When you can't explain or pinpoint exactly why, it's hard to get people to believe it's happening. I think one of the most troubling aspects is the ice melting and pictures of starving polar bears. I think these pictures could make people see the damage being done.
So many varying opinions. Is it just the natural curve in temperature.
The confusion between "global warming" and "climate change"
Differing theories
We don't know what's causing it
That people don't believe it's real.
prevention
HOW MUCH OCCURS NATURALLY VS HOW MUCH HUMANS CAUSE
The most confusing part is if to much or too little affects the earth.
It troubles me that it has become "political". It makes the news untrustworthy.
Hot the weather keeps getting hotter and we barely had a winter this year.
that people make it a bigger deal then need be
I may not understand all the science of global warming, but I realize the importance of addressing it. I fail to understand what possible motive climate change scientists and supporters could have to push a false idea of global warming. Climate change deniers don't make any sense to me. I understand why rich people deny climate change, but how do they convince so many poor people to support them when the poor people have nothing to gain and so much to lose? As far as the science is concerned, I don't really know enough about it to comment on what confuses me. I know what behaviors are harmful to Earth and it makes total sense to me that a decent person would just abide by the simple guidelines scientists have proposed for every day people to help out.
I dont know much about it so I would say just about all of it. Its not that I don't, I have 3 children so keeping up with stuff outside of school related things is not always possible
How there is an entire school of thought that truly believes there is no such thing as global warming and it is all a hoax perpetuated by environmental conservationists. And the result is that these powerful and influential "non-believers" are turning average people with little education or information on the subject into non-believers as well. It is baffling and terrifying.
It is a large effect on the earth.
Lies and poisons being fed to confuse people. TRUTH laid out with no secrets is much easier to comprehend and fix issues.
It is very complex, and I have not taken any science classes beyond high school, so it is just generally difficult and complicated to understand.
it's implications for future generations
Not a lot of people believe in it
There seems to not be enough definitive information for/against global warming theory to convince everyone.
The confusing part is that there are so many sides to be taken about something that should be a true or false situation. There are so many different possible causes and people so often fixate on one issue. A lot of people aren't sure what to believe because there are so many opinions and so little agreement or convincing facts shared.
The most trouble aspect is how few people understand (or "believe") in it and feel that action needs to be taken.
The most troubling aspect to global warming to me is that people listen to political spin on it instead of getting facts and educating themselves on it.
It bothers me that the whole global warming issue is politically motivated. It is hard to believe the global warming rhetoric when it is spouted by leftist celebrities who do not practice what they preach.
How to fix the damage that has already been caused
There seems to be some uncertainty if it is caused entirely by humans or is a natural cycle of the Earth's climate, since we have limited historical data.
melting of glaciers and change in oceanic temperature
I think global warming is caused by geo-engineering.
The whole concept of it.
the terms used are confusing
Rising sea levels are the most troubling aspect, as this would affect many coastal cities all over the world.
There isn't US leadership supporting science and global warming.
No one trusts the news media anymore so very hard to get facts relating to this
Not sure.
It is all very confusing to me. I have not followed global warming so I do not know very much about it at all.
The most confusing and troubling aspect is that most Republicans don't believe in it. How can they not take this issue seriously?
most troubling is that the earth may become uninhabitable for my species
How to explain it clearly and efficiently to people who don't understand the concept.
Although I believe that global warming is mostly caused by humans, I also believe that a portion of global warming is a natural progression.
How to help fix it as a community/
I don't feel we have enough long term information to identify any real global warming.
Living in a coastal.state (Florida), I'm most troubled by the implications of rising ocean levels and beach erosion.
How to truly get a change to happen to decrease the global warming
How they can determine what it causing it.
How nobody believes that global warming is actually happening.
That everyone seems to have an agenda with global warming so it's hard to know what is true
The people who deny it is happening
how some people still claim it doesn't exist.
Though loss of life, the most troubling thing is the intense division among people to believe in (or deny) and rectify (or not) the situation. We're THE ONLY ONES who can save the planet…which basically makes us superheroes. But we're stuck vacillating over whether a threat exists…and treating each other horribly because of it. I think even most global warming deniers can agree that we should take care of the Earth. I wish we could just see climate change efforts as taking care of the Earth instead getting bogged down in why we're saving the Earth. It's the freakin' Earth!
I would have to say, the fear of the possibility of icebergs melting, causing the sea levels to rise.
The lack of data on potential solutions -- the problem seems to be very well characterized, but the general public has very little idea what to do about it.
Determining fact or fiction from media and politicians.
They say global warming, then it's the coldest winter ever…then they change it to "climate change". Climate always changes, we can't do anything about it.
I have never heard of greenhouse gases or aerosol effects. They are slightly confusing to me.
What I personally can do about it.
That people don't believe it is happening.
What or who is responsible
Mixed Information
That Western countries, that have stronger control over emissions are penalized for pollution, but 3rd World countries, such as China, India and Russia, major contributors of pollution that don't have strict control over emissions, are not penalized for pollution
How it's not accepted as valid science by many
It troubles me that the cattle industry causes an enormous output of greenhouse gases and yet this fact is largely hidden from the American public due to the monetary influence of special interest groups.
I am confused as to why governments are spending millions of dollars on global warming. I feel like global warming is going to occur with the advancement of technology
The most troubling aspect is that so many people refuse to acknowledge something that such a high percentage of scientists agree on that is so dangerous for the entire planet, including themselves.
I am not confused, but I am concerned about global climate change.
We know what things contribute to it but not what exactly what it will cause to happen in the future to Earth.
What really causes it
I haven't read too much into this subject
That people are solely responsible for climate change and global warming.
fear tactics
Humans
how did climate change- able to stop
The earth's delicate ecosystem
How it started.
That the politicians don't care about Global Warming and are not looking for solutions.
Our extinction
Mixed message and historical data on previous ice ages
ocean levels rising
The most troubling aspect related to global warming is the simplistic ease which writers on the internet, or on the mainstream media news loudly declare daily that the care of the planet Earth is the only thing that people should care about, sacrifice for, and pay for; these writers/pontificators ASSUME that all of their information is correct and use no in-depth fact checking at all. The old phrase "The sky is falling….prepare to die" springs to mind. Sad.
how it is causing extreme weather disasters
smog carbon dioxide cause a lot killing of the vegetation on the earth.
That it could kill us
So many different researchers given different answers and predictions
What to do about it.
very
What is causing and how it can be slowed
The most troubling aspect of global warming for me, is the melting of polar ice-caps. Rising water levels and salinity changes may have negative effects on the environment for humans and wildlife. While the earth naturally goes through changes, it is those that disrupt human survival that is the most scary for us.
I hear news about the global temperature increasing, but have never seen the hard data for the global data.
The most troubling is the fact that although we as a people know that global warming exists, we still have not done enough to stop it. The human footprint on the earth is destroying the earth. We as a people have been the most destructive to the planet…. and won't do anything about it until its too late.
How we affect our environment and the best way to fix the issue. There are so many studies and opinions it's hard to decipher.
what is most confusing and/or troubling is the fact that no one can seem to clearly identify specifically what is cause our earth to become more and more uninhabitable and by no means can any of our scientists seem to agree on anything together.
I feel that there is not enough research out there on this subject and those that are researching from both sides are not coming together to help each other in this problem that we have created.
How it began is not clear or when.
THE MOST CONFUSING PART FOR ME IS THAT EVERYTHING THAT IS BEING ECPLAINED I CANNOT SEE SO IS VERY HARD TO BELIEVE
I'm not sure if It's actually, "global warming" Too many other factors in place.
Mars is warming, and you can't blame that on human activity. The NOAA and NASA are altering real world data and historic data to fit their models instead of altering the models to fit the "pause". Climate change was the new name of global warming when we stopped warming, and right now, its main purpose is to justify totalitarian control of resources and population because it covers everyone instead of "for the children" when a fifth don't have children. It is modern Lysenkoism, liberals using politicized science to justify political policy.
what can be done to decrease the global temp
it seems very politically driven and is hard to decipher the truth
The uncertainty of what kind of planet we are leaving to our children
Why it is happening
How to correct it or make it better
Propaganda
none
Who and what do we believe. The answers and cause and effect seem to change every so often. It is very confusing if we are not given the correct information to stay on top of it.
All the different opinions, so that you don't know who to believe
rising sea levels; changing habitat of microbes and the animals that carry them
It's killing a lot of wild life and other living things.
The fact that people like Al Gore and other politicians and others got millions of dollars from this big lie.
whether it is a naturally occurring phenomenon or if humans are causing it
That people don't believe it's happing and don't want to make things better.
going to get to hot to live on earth
All the conflicting stories and "facts" are confusing.
Ignorance of others. There is nothing confusing about it, it's just other people don't understand basic science so they refute things because they don't/ can't understand them.
Anthropogenic causes mixing with the Earth's natural cooling and warming cycle, are humans fully to blame? Or just mostly?
all chemicals in air
people who don't believe it's an issue
livestock is killing our resources
How little things make such a big impact
There is no real consensus within the Scientific community. The theories seem to change every so often to reflect the political climate of the time. It also changes as theories from 10–20 years ago are proven false.
Some people provide "facts" that it isn't real and others provide "facts" that it is real. So what is the hard, science-based truth?
I read many different articles from credible sources that we have entered the point of no return in regards to climate change.
What's troubling is human's need to burn so much oil and generate so much pollution.
The most troubling aspect is that there are still people out there that do not believe that global warming is happening. I think the scientific community needs to put out more reader friendly information about their findings for the general community.
How to control it
The name "global warming is misleading. I find the term climate change is more accurate
Why are the winters so cold if global warming is an issue? Would the earth still be warming without global warming? Is it realistic to reduce human impact on earth? How long will it be before real problems happen? Years from now or millions of years from now? How does stopping global warming affect the economy? Is trying to stop global warming a real thing?

## 2 Experimental design, materials and methods

The 20-question survey was composed by the author of this report and submitted to the Texas Tech Institutional Review Board for approval to be implemented. Such approval was granted on March 21, 2017 for IRB2017-282 “Online Survey to Collect Opinions about Factors Influencing Global Warming.” The online survey instrument was created within Google forms and is available at https://goo.gl/forms/rfuRKw5jT37raWmf1. Participants were recruited by three methods. First social media (Facebook) posts and ads were used to encourage survey completions with no monetary reward to participants. Secondly, a Texas Tech University daily announcement email (TechAnnounce) was used to recruit members of the University community. Lastly, Amazon Mechanical Turk was used to recruit participants. Individuals who completed the survey through Mechanical Turk were rewarded with $0.05–0.10 per survey completion. Recruiting participants through Mechanical Turk was by far the most effective means, and roughly 600 of the surveys 746 respondents replied via Mechanical Turk. At the completion of the polling period, data was downloaded from the Google forms site for reporting.

