# Supplementary material for: Survey data reflecting popular opinions of the causes and mitigation of climate change
Source: Data Brief. 2017 Jul 27;14:412–39. doi: 10.1016/j.dib.2017.07.060 (PMC5552377; doi:10.1016/j.dib.2017.07.060)
Supplement: Supplementary file 1 — Supplementary material [file mmc1.docx]

**Conflict of Interest Form**

The author declares no competing conflicts of interest exist in regard to the publication of this article.
